# CSF biomarker analysis of *ABCA7* mutation carriers suggests altered APP processing and reduced inflammatory response

**DOI:** 10.1186/s13195-023-01338-y

**Published:** 2023-11-09

**Authors:** Lena Duchateau, Fahri Küҫükali, Arne De Roeck, Mandy M. J. Wittens, Joke Temmerman, Ilse Weets, Maarten Timmers, Sebastiaan Engelborghs, Maria Bjerke, Kristel Sleegers

**Affiliations:** 1 Complex Genetics of Alzheimer’s Disease Group, VIB-UAntwerp Center for Molecular Neurology, VIB, Building V, Universiteitsplein 1, Wilrijk, Antwerp B-2610 Belgium; 2 Department of Biomedical Sciences, University of Antwerp, Universiteitsplein 1, Wilrijk, Antwerp 2610 Belgium; 3 Present Address: Argenx, Ghent, Belgium; 4 Clinical Neurochemistry Laboratory, Department of Clinical Biology, University Hospital Brussels, Generaal Jacqueslaan 137, Elsene, Brussels 1050 Belgium; 5 Neuroprotection & Neuromodulation (NEUR) Research Group, Center for Neurosciences (C4N), Vrije Universiteit Brussel (VUB), Laarbeeklaan 103, Jette, Brussels 1090 Belgium; 6 Experimental Pharmacology (EFAR) Research Group, Center for Neurosciences (C4N), Vrije Universiteit Brussel (VUB), Laarbeeklaan 103, Brussels, 1090 Belgium; 7 Reference Center for Biological Markers of Dementia, Department of Biomedical Sciences, University of Antwerp, Universiteitsplein 1, Wilrijk, Antwerp 2610 Belgium; 8 Janssen Research and Development, a Division of Janssen Pharmaceutica NV, Turnhoutseweg 30, Beerse, 2340 Belgium; 9 Department of Neurology and Bru-BRAIN, University Hospital Brussels, Generaal Jacqueslaan 137, Elsene, Brussels 1050 Belgium

**Keywords:** ABCA7, PTC mutation, VNTR expansion, CSF biomarkers, Alzheimer’s disease, Inflammation, Amyloid-β, Tau

## Abstract

**Background:**

The Alzheimer’s disease (AD) risk gene *ABCA7* has suggested functions in lipid metabolism and the immune system. Rare premature termination codon (PTC) mutations and an expansion of a variable number of tandem repeats (VNTR) polymorphism in the gene, both likely cause a lower *ABCA7* expression and hereby increased risk for AD. However, the exact mechanism of action remains unclear. By studying CSF biomarkers reflecting different types of AD-related pathological processes, we aim to get a better insight in those processes and establish a biomarker profile of mutation carriers.

**Methods:**

The study population consisted of 229 AD patients for whom CSF was available and *ABCA7* sequencing and VNTR genotyping had been performed. This included 28 PTC mutation and 16 pathogenic expansion carriers. CSF levels of Aβ_1–42_, Aβ_1–40_, P-tau_181_, T-tau, sAPPα, sAPPβ, YKL-40, and hFABP were determined using ELISA and Meso Scale Discovery assays. We compared differences in levels of these biomarkers and the Aβ ratio between AD patients with or without an *ABCA7* PTC mutation or expansion using linear regression on INT-transformed data with *APOE*-status, age and sex as covariates.

**Results:**

Carriers of *ABCA7* expansion mutations had significantly lower Aβ_1–42_ levels (*P* = 0.022) compared with non-carrier patients. The effect of the presence of *ABCA7* mutations on CSF levels was especially pronounced in *APOE* ε4-negative carriers. In addition, VNTR expansion carriers had reduced Aβ_1–40_ (*P* = 0.023), sAPPα (*P* = 0.047), sAPPβ (*P* = 0.016), and YKL-40 (*P* = 0.0036) levels.

**Conclusions:**

Our results are suggestive for an effect on APP processing by repeat expansions given the changes in the amyloid-related CSF biomarkers that were found in carriers. The decrease in YKL-40 levels in expansion carriers moreover suggests that these patients potentially have a reduced inflammatory response to AD damage. Moreover, our findings suggest the existence of a mechanism, independent of lowered expression, affecting neuropathology in expansion carriers.

**Supplementary Information:**

The online version contains supplementary material available at 10.1186/s13195-023-01338-y.

## Background

First identified in a genome-wide association study (GWAS) on Alzheimer’s disease (AD), ATP-binding cassette transporter family A member 7 (*ABCA7*) is now widely established as a risk gene for AD [1–4]. Besides common risk variants, rare variants in *ABCA7* have been found at increased frequencies in AD patients, most notably rare premature termination codon mutations (PTC), which are found throughout the gene [5–9]. In addition, an expansion of a variable number of tandem repeats (VNTR) polymorphism, located in intron 18 of the gene, is associated with AD [9]. PTC mutations and the VNTR expansion result in a stronger increase in AD risk (2.6-fold and 4.5-fold, respectively) than the common variants identified in GWAS [10]. Both variants have been found to lower *ABCA7* dosage and are thought to alter AD risk through haploinsufficiency [5, 7, 9]. Moreover, even without a VNTR expansion, an increase in length of the VNTR alleles is associated with lower *ABCA7* expression [9]. *ABCA7* encodes for a lipid transporter and is thought to play a role in lipid metabolism, where it mediates phospholipid export, and phagocytosis by microglia, and thus affecting immune regulation [10, 11]. Moreover, *ABCA7* PTC mutation carriers often suffer from cerebral amyloid angiopathy (CAA) [12]. However, the physiological function of ABCA7 has not yet been fully elucidated nor is it clear through which exact mechanisms of action ABCA7 reduction could result in AD. A better understanding of the risk-increasing pathways and underlying neuropathology is vital to better understand the disruptive processes leading to AD and to ultimately aid in finding treatment for *ABCA7* mutation carriers.

One way to get insight into these pathways is to use biomarkers reflecting pathways specifically associated with AD or AD-associated processes. Cerebrospinal fluid (CSF) biomarkers are already used in both research and clinical environments to identify, predict, and investigate AD [13, 14]. To ameliorate our understanding of the in vivo pathways affected by *ABCA7* mutations, we here investigated nine CSF biomarkers, reflecting different pathological processes, in a unique cohort of mutation and/or VNTR expansion carriers. These include amyloid-β_1–42_ (Aβ_1–42_), amyloid-β_1–40_ (Aβ_1–40_), the Aβ_1–42_/Aβ_1–40_ (Aβ ratio), and α and β cleaved soluble amyloid precursor protein (sAPPα and sAPPβ) to investigate a potential role of ABCA7 in Aβ deposition and APP processing [13, 15–17]. Total tau (T-tau) and phosphorylated tau at threonine 181 (P-tau_181_) CSF biomarkers are indicative of neurodegeneration and formation of neurofibrillary tangles (NFT), respectively [13, 15]. In addition, we selected chitinase-3-like protein 1 (CHI3L1; also known as YKL-40) to investigate a role of ABCA7 in glial activation and neuroinflammation and heart fatty acid binding protein (hFABP) which further reflects neurodegeneration [13, 16, 18, 19]. For each of these pathological processes, ABCA7 has been hypothesized to play a role. Several in vitro and in vivo studies have indicated that a reduced *ABCA7* expression may exacerbate amyloid pathology by affecting either APP processing or amyloid clearance [20–24]. Through its possible functions in microglia and phagocytosis, ABCA7 could also influence inflammation. Finally, for neurodegeneration and tangle formation, there is less of a direct connection to ABCA7 function, though through its functions in inflammation and plaque formation, ABCA7 could impact neuronal loss, and plaque load has been suggested to impact tangle development in several studies [25]. The inclusion of the Aβ ratio, P-tau_181,_ and T-tau biomarkers moreover allowed for adding an ATN-classification [26].

## Methods

### Study population

CSF samples were available of 229 AD patients and 64 healthy control individuals (Additional file 1: Table 1). Patients were ascertained from the ZNA Memory Clinic, Antwerp, Belgium, and were diagnosed with possible, probable, or definite AD by at least two neurologists based on the National Institute on Aging—Alzheimer’s Association diagnostic criteria [27]. *ABCA7* sequencing had previously been performed [5]. Samples were selected in our Belgian cohort based on the presence of VNTR length data or presence of a PTC mutation, as well as access to CSF biomaterial of the patient. Of the 229 AD patients, 28 carried an *ABCA7* PTC mutation. VNTR lengths had been genotyped previously by Southern blotting for 212 of the 229 patients and had aid in identifying 16 expansion carriers (allele length > 5720 bp or 229 repeats) [9]. The patient group included 17 PTC mutation carriers for whom VNTR length could not be determined. One patient carried both a PTC mutation and a VNTR expansion. Information on the demographics of the *ABCA7* mutation carriers can be found in Additional file 1: Table 5. Within the group of AD patients, CSF Aβ-ratio was used to distinguish between individuals with in vivo evidence of Aβ-positivity (A+ < 0.12) and those who were Aβ-negative. The Aβ-positive group contained 163 patients (23 PTC carriers and 13 expansion carriers), and Aβ-negative group consisted of 66 patients (4 PTC carriers, 2 expansion carriers and 1 carrier with both a PTC mutation and an expansion) (Table 1 and Additional file 1: Table 1). Moreover, we applied ATN classification, with A based on Aβ positivity as described above, T concerning tangle pathology represented by CSF P-tau_181_ (T+ > 57 pg/mL) and N depicting neurodegeneration characterized by CSF T-tau (N+ > 297 pg/mL) [26]. ATN classification of the *ABCA7* mutation carriers can be found in Additional file 1: Table 5.

**Table 1:** Aβ-positive cohort's demographics and biomarker levels

	**Mutation carriers**			**Non-carriers AD**
	**All**	**PTC carriers**^**a**^	**VNTR expansion carriers**^**a**^	
Samples (*n*)				
Aβ+	36	23	13	127
AAO	72.9 (10.3)	71.3 (10.9)	75.8 (8.7)	75.6 (8.1)
Sex				
Male	14 (38.9%)	10 (43.5%)	4 (30.8%)	45 (35.4%)
Female	22 (61.1%)	13 (56.5%)	9 (69.2%)	82 (64.6%)
APOE ε4				
Positive	24 (66.7%)	16 (69.6%)	8 (61.5%)	75 (59.1%)
Negative	12 (33.3%)	7 (30.4%)	5 (38.5%)	52 (40.9%)
Biomarkers (*n*)				
Aβ_1–42_ (pg/mL)	490.5 (273.3)	535 (345.5)	385 (228)	578 (308)
Aβ_1–40_ (pg/mL)	5957.5 (3734.3)	6638 (4086.5)	4405 (1431)	6738 (3088.5)
Aβ ratio	0.089 (0.033)	0.081 (0.026)	0.09 (0.04)	0.09 (0.03)
sAPPα (ng/mL)	97.5 (66.3)	119 (85.5)	83 (27)	113.5 (74.8) (*n* = 126)
sAPPβ (ng/mL)	93.5 (72.5)	110 (84.5)	82 (20)	117 (83)
YKL-40 (ng/mL)	187 (90)	208 (80)	150 (45)	201 (112.5)
hFABP (ng/mL)	4.39 (2.8)	4.86 (2.37)	3.2 (2.9)	4 (1.8) (*n* = 126)
T-tau (pg/mL)	619 (544) (*n* = 29)	723 (386) (*n* = 17)	464 (475.5) (*n* = 12)	548 (428) (*n* = 112)
P-tau_181_ (pg/mL)	70 (53) (*n* = 29)	76 (45) (*n* = 17)	60.5 (57.8) (*n* = 12)	81 (47.5) (*n* = 111)

Demographics are presented as mean (SD) or mean (%). Non-parametric biomarker levels are presented as median (interquartile range). If biomarker data was not available for the entire subgroup, the number of samples included is shown

^a^One sample had both a PTC mutation and VNTR expansion and was included in both subgroups

CSF of Aβ-negative control individuals (CDR 0) was derived from participants of study 54861911ALZ1005 (NCT01978548) [28] and 54861911ALZ2002 (NCT02260674) [29] who screen failed due to being Aβ_1–42_ negative (CSF Aβ_1–42_ > 600 ng/L). DNA for genetic analysis was not available for these control individuals, and thus, this cohort is only used for reference of physiological levels. All participants, and/or their legal guardians, signed a written informed consent and study protocols were approved by the ethics committee of University of Antwerp/Antwerp University Hospital.

### Biomarker analyses

CSF was obtained by lumbar puncture (LP) at the L3/L4 or the L4/L5 interspace [30]. A minimum of 2 ml CSF was collected for each patient in a labeled polypropylene tube. In case of a hemorrhagic puncture detected by macroscopic inspection of the sample, samples were centrifuged for 10 min at 3000 rpm within 4 h after LP. After centrifugation, the supernatant was transferred to a new, labeled polypropylene tube. Samples were either frozen immediately in liquid nitrogen and shipped on dry ice to our biobank or shipped on wet ice within 24 h after LP. Samples were stored in the biobank at −80°C until analysis. Control samples were processed in a similar way according to industry standards as detailed previously [28, 29]. Both AD and control samples were thus processed adhering to the Alzheimer’s Association international guidelines to minimize variance in pre-analytical procedures and bias [31]. For both the clinical and the control cohorts, the CSF Aβ_1–42_ and Aβ_1–40_ were analyzed using commercially available enzyme-linked immunosorbent assays (ELISA; EUROIMMUN, Lübeck, Germany) on the fully automated Analyzer I-2P (EUROIMMUN, Lübeck, Germany) according to manufacturer’s protocol. The Aβ_1-42_ (normal range > 824 pg/mL) and Aβ_1-42_/Aβ_1-40_ (ratio; normal range > 0.12) cut-offs were determined in house based on clinical and autopsy confirmed cohorts consisting of AD patients and healthy subjects at the Reference Center for Biological Markers of Dementia, Department of Biomedical Sciences, University of Antwerp. For the AD patients, T-tau and P-tau_181_ were determined using commercially available INNOTEST ELISA kits according to the instructions of the manufacturer (Fujirebio, Ghent, Belgium). Previously published cut-offs (normal range T-tau < 297 pg/mL and P-tau181 < 57) were applied for the ATN classification [30]. For the control subjects, T-tau and P-tau181 were assessed using the same INNOTEST assays as for the AD patients (Fujirebio, Ghent, Belgium), while the analyses were performed at the Sahlgrenska University hospital, Molndal, Sweden. For the AD patient and healthy subjects, hFABP, sAPPα, and sAPPβ were measured by commercially available electrochemiluminescent (ECL) ELISAs (Meso Scale Discovery (MSD), MD, USA), while YKL-40 was analyzed with a commercially available ELISA (R&D systems, Inc., Minneapolis, USA) at the Clinical Neurochemistry laboratory at the University Hospital Brussels; all in accordance with manufacturer’s instructions. For all the above assays, the intra-assay coefficient of variation (CV) ranged from 1.4 to 3.0% and the inter-assay CV from 6.4 to 11.2%.

### Statistical analysis

Participant characteristics (age, sex, and *APOE* ε4 positivity) were compared between AD patient groups using Kruskal–Wallis (for age) and *χ*^2^ statistics (for sex and *APOE*). Normality of the biomarkers was assessed using normal quantile–quantile plots and the Shapiro–Wilk test. None of the biomarkers followed a normal distribution. Thus, either non-parametric tests were performed or data was transformed using rank-based inverse normal transformation (INT) followed by parametric testing. Differences in biomarker means between AD patients and controls were assessed using a Mann–Whitney *U* test. Absolute T-tau and P-tau_181_ values were not compared as they were analyzed at different laboratories for the AD patients and controls subjects. Within the group of AD patients, separate linear regressions were performed to compare biomarker concentrations among different genetic groups: (1) *APOE* ε4 positive subjects compared with negative, (2) mutation carriers compared with non-carriers, (3) PTC carriers alone compared with non-carriers, (4) expansion carriers alone compared with non-carriers, and (5) with sum of VNTR alleles length as continuous variable (with carriers of PTC mutations excluded). Mutation carriers were defined as subjects having a PTC and/or VNTR expansion mutation. The individual with both a VNTR and PTC mutation was included in both groups for analysis. Age and sex were analyzed in a linear regression together. For *APOE* ε4, age and sex were included as covariates. For the other three comparisons, age, *APOE* ε4 status (ε4-positive or ε4-negative), and sex were included as covariates. All analyses were performed on the AD cohort only, as no genetic data were available for the control subjects. The clinically diagnosed AD cohort included 66 Aβ-negative individuals. Among those, 14 had Aβ-ratio values close to the cut-off (between 0.120 and 0.125), nine had mean Aβ-ratio of 0.134 with abnormal Aβ_1-42_ and T-tau/p-tau values, and two received postmortem diagnosis of definite AD, suggesting that at least some of the Aβ-negative cases were true AD patients. We decided to perform all primary analyses on the Aβ-positive cohort; additional analyses on the full cohort including Aβ-negative cases are presented in the additional tables and figures. As both an expanded VNTR and PTC mutations are expected to have a dosage reducing effect on *ABCA7* expression, they were studied together as well as separately. Participants missing biomarker data were omitted from the respective linear regression model (Table 1 and Additional file 1: Table 1). Results are reported as β-regression coefficients with standard errors (SE) and *P*-values. Differences between biomarker levels of *APOE* ε4-positive and ε4-negative samples, further stratified according to *ABCA7* mutation or expansion carrier status, were assessed with a Mann–Whitney *U* test. All analyses were performed using R, version 3.6.2.

Power calculations were performed using the pwr package in R. In the group of Aβ-positive carriers, for mutation carriers (*n* = 36), PTC carriers (*n* = 23), and expansion carriers (*n* = 13) respectively differences of Cohen’s *d* ≥ 0.5, *d* ≥ 0.6, and *d* ≥ 0.8 can be detected at alpha = 0.05 and 80% power. The full dataset has 84% power to detect differences of medium effect size (Cohen’s *d* = 0.5) at alpha = 0.05 when comparing the total group of mutation carriers (*n* = 43) to non-carriers (*n* = 186).

## Results

In this study, CSF biomarkers were investigated in a cohort of 199 Aβ-positive AD patients, among whom 23 *ABCA7* PTC carriers and 13 VNTR expansion carriers. Analysis on the full cohort of 229 clinically diagnosed AD patients carriers and 64 control subjects is presented in the Additional Figures. Demographics and CSF biomarker data are shown in Table 1 and Additional file 1: Table 1. Information about clinical diagnosis, age at onset (AAO), familial history, ATN-classification, *APOE* status, type of mutation, and disease duration (DD) at LP of *ABCA7* mutation carriers can be found in Additional file 1: Table 5. Significantly decreased concentrations of Aβ_1–42_ (*P* < 0.001), Aβ_1–40_ (*P* = 0.018), as well as a lower Aβ ratio (*P* < 0.001), sAPPα (*P* < 0.001), and sAPPβ (*P* < 0.001) were found in AD patients compared with controls, while YKL-40 (*P* < 0.001) and hFABP (*P* < 0.001) were found to be increased in AD patients.

### Biomarkers reflecting APP processing and amyloid pathology

**Fig. 1:**
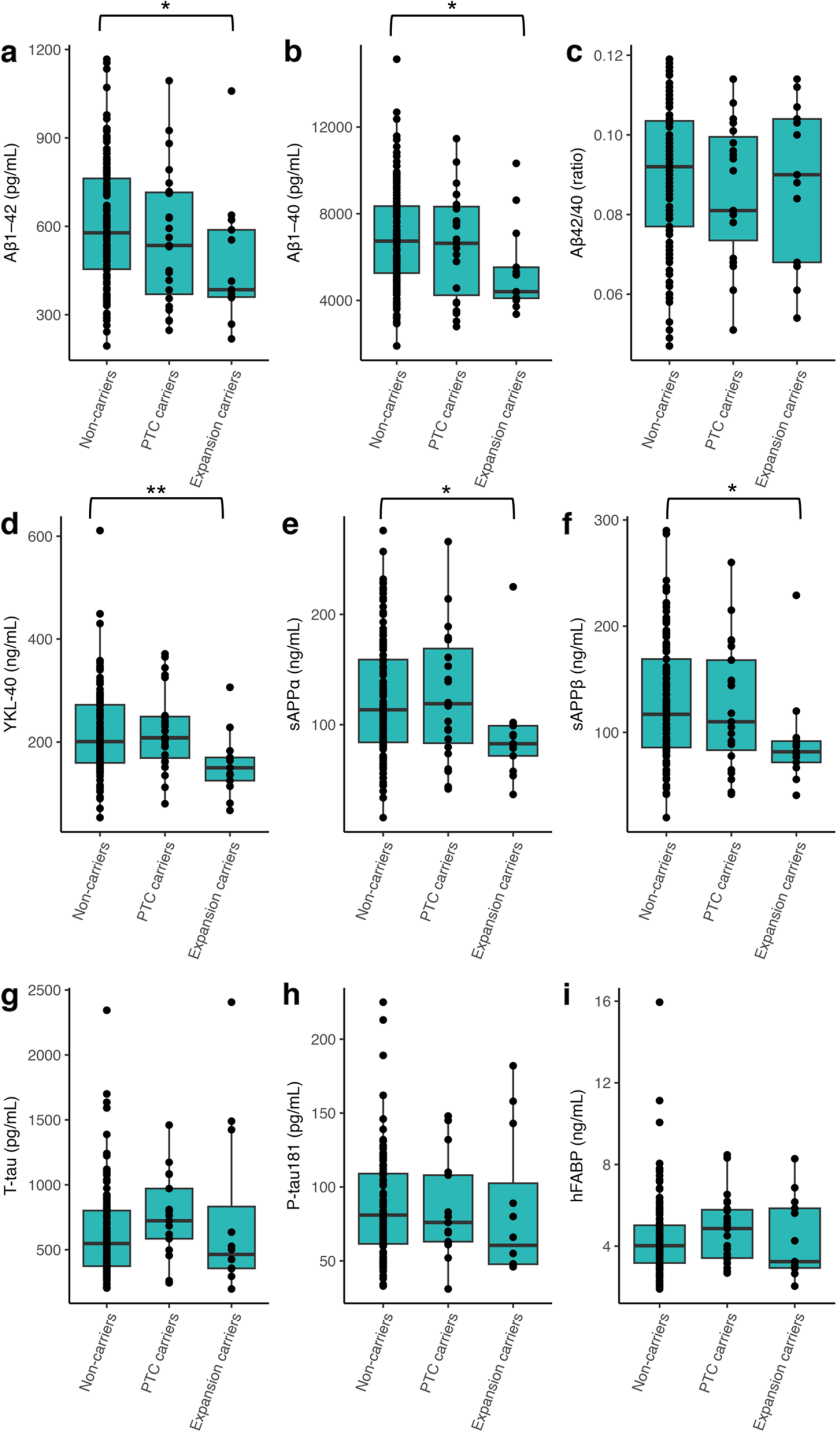
CSF biomarker levels in different study groups in the Aβ-positive cohort. Comparison of different groups of *ABCA7* mutation carriers or non-carriers, in the Aβ-positive cohort, using boxplots, depicting median and IQR, for the untransformed biomarkers: Aβ_1–42_ (**A**), Aβ_1–40_ (**B**), Aβ ratio (**C**), YKL-40 (**D**), sAPPα (**E**), sAPPβ (**F**), T-tau (**G**), P-tau_181_ (**H**), and hFABP (**I**). Linear regression on INT transformed data was performed between different groups with age, sex, and *APOE* ε4 status as covariates. *P < 0.05, **P < 0.01

**Fig. 2:**
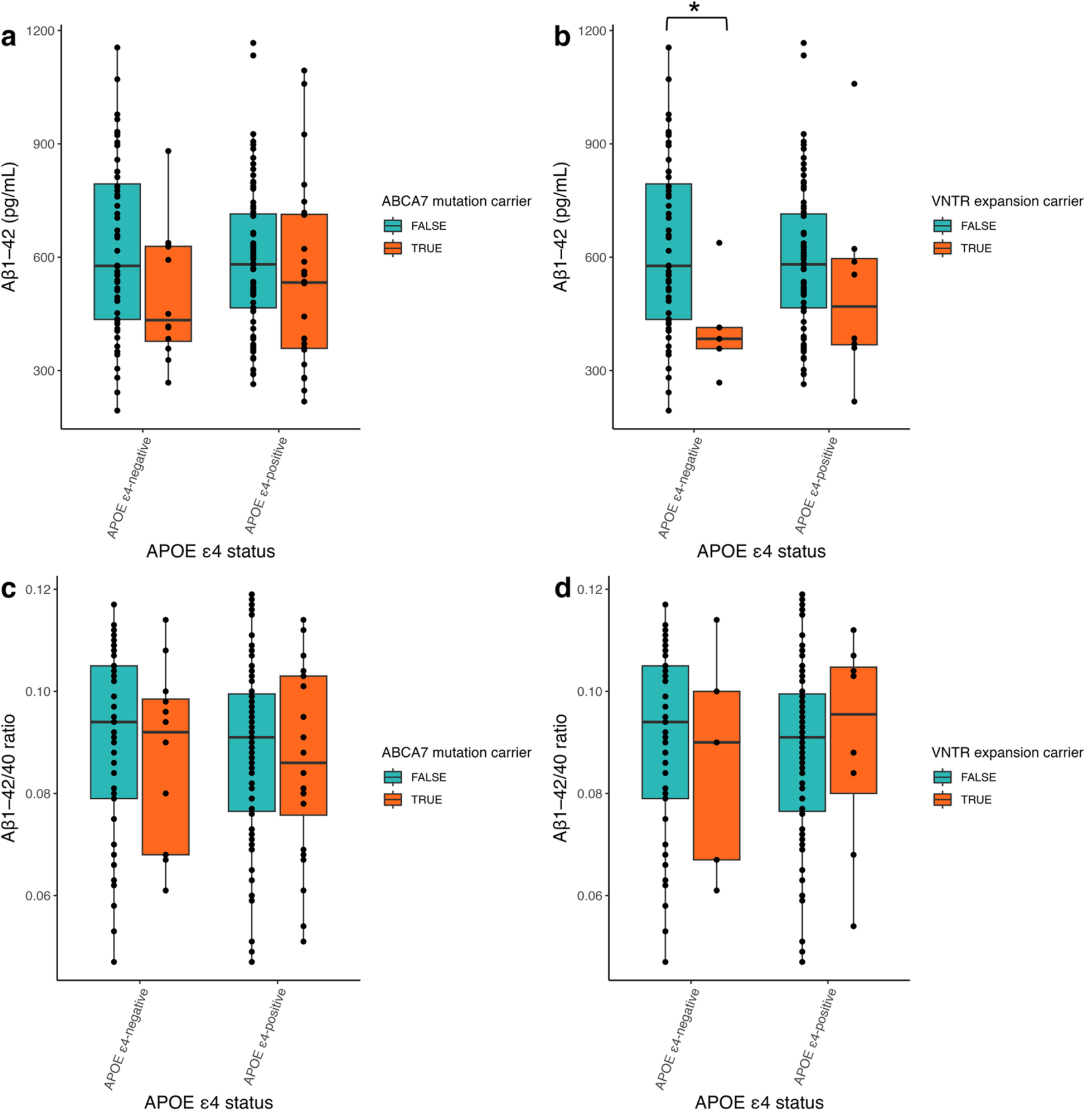
Aβ_1–42_ and Aβ ratio levels along *APOE* ε4 and *ABCA7* carrier status in the Aβ-positive cohort. Median Aβ_1–42_ (**a**, **b**) and Aβ ratio (**c**, **d**) levels according to *APOE* ε4 status and further stratified for *ABCA7* mutation carrier status (**a**, **c**) or VNTR expansion carrier status (**b**, **d**) in the Aβ-positive cohort. Significance was assessed with a Mann–Whitney *U* test on untransformed data. *P < 0.05

The association between mutation status and CSF biomarkers was investigated within the group of AD patients with in vivo evidence of amyloid pathology, between carriers of the different *ABCA7* mutations and non-carriers. It was further re-assessed in the full group of all clinically diagnosed AD patients. The CSF levels of Aβ_1–42_ were significantly decreased in expansion carriers in the Aβ-positive cohort (median_ABCA7+_ = 385 pg/mL, *P* = 0.022) when compared with non-carriers (median_ABCA7-_ = 578 pg/mL) (Table 2, Fig. 1). The same observation could be made in the full cohort (median_expansion+_ = 399.5 pg/mL, median_ABCA7-_ = 618 pg/mL, *P* = 0.0022). Moreover, in the full cohort, Aβ_1–42_ CSF levels were significantly decreased in carriers with *ABCA7*-reducing mutations (either PTC or VNTR expansion carriers) (median_ABCA7+_  = 535 pg/mL, *P* = 0.014) (Additional file 1: Table 3, Additional file 2: Fig. 2). The Aβ-positive cohort was further stratified for *APOE ε4* and *ABCA7* mutation status, and we found significantly lower levels of Aβ_1–42_ in ε4 negative carriers with an expansion mutation (median_APOE4-/expansion+_  = 384 pg/mL, median_APOE4-/expansion-_ = 577 pg/mL, *P* = 0.031), but no significant differences for *ABCA7* mutations or when considering Aβ ratio (Fig. 2). In the full cohort, *APOE* genotype has an impact on Aβ_1–42_ levels (median_APOE4+/ABCA7-_ = 583 pg/mL, median_APOE4-/ABCA7-_ = 733 pg/mL, *P* = 0.0014) (Additional file 1: Table 4). In ε4 negative carriers, having an *ABCA7* mutation further reduced the Aβ_1–42_ biomarker concentrations (median_APOE4-/ABCA7+_  = 547 pg/mL, median_APOE4+/ABCA7+_  = 535 pg/mL, *P* = 0.02), in which the Aβ_1-42_ concentration was similar to that of ε4 allele carriers (Additional file 2: Fig. 4). This trend also remained when looking at expansion carriers alone (median_APOE4+/expansion+_  = 554 pg/mL, median_APOE4-/expansion+_  = 384 pg/mL with *P* = 0.0019), but not when considering Aβ ratio (Additional file 2: Fig. 4).

**Table 2:** Associations between CSF biomarkers and *ABCA7* mutation status in the Aβ-positive AD cohort

	**Mutation carriers vs non-carriers**								
	**All**			**PTC carriers**			**Expansion carriers**		
	***β***	**SE**	***P*-value**	***β***	**SE**	***P*-value**	***β***	**SE**	***P*-value**
Aβ_1–42_	-0.31	0.16	0.065	-0.15	0.2	0.46	-0.57	0.25	*0.022*
Aβ_1–40_	-0.28	0.18	0.13	-0.069	0.22	0.76	-0.63	0.27	*0.023*
Aβ ratio	-0.085	0.14	0.53	-0.12	0.16	0.46	-0.033	0.21	0.88
sAPPα	-0.18	0.2	0.37	0.067	0.24	0.78	-0.59	0.29	*0.047*
sAPPβ	-0.3	0.19	0.11	-0.068	0.23	0.77	-0.69	0.28	*0.016*
YKL-40	-0.14	0.18	0.44	0.24	0.22	0.26	-0.79	0.27	*0.0036*
hFABP	0.21	0.18	0.23	0.37	0.21	0.084	-0.056	0.27	0.84
T-tau	0.18	0.19	0.34	0.32	0.23	0.17	0.01	0.27	0.97
P-tau181	-0.11	0.18	0.53	-0.06	0.22	0.79	-0.18	0.26	0.5

Linear regression on INT transformed biomarker data performed between the shown groups with age, sex, and APOE ε4 status as covariates. Mutation carriers include both PTC and VNTR expansion mutation carriers. Significant *P*-values (*P* < 0.05) are shown in italics

The Aβ_1–40_ concentration was only significantly different in VNTR expansion carriers (Aβ-positive cohort: median_expansion+_  = 4405 pg/mL, full cohort: median_expansion+_  = 4397 pg/mL), where it was lower than in non-mutation carriers (Aβ-positive cohort: median_ABCA7-_ = 6738 pg/mL, *P* = 0.023; full cohort: median_ABCA7-_ = 6099.5 pg/mL, *P* = 0.016 respectively) (Table 2, Fig. 1; Additional file 1: Table 3, Additional file 2: Fig. 2). In the Aβ-positive cohort, Aβ_1–40_ levels slightly increased with increasing age (Additional file 1: Table 2). When combining the previous two markers into the Aβ ratio, carriers of a PTC mutation and/or VNTR expansion was not changed in either cohort (Table 2, Additional file 1: Table 3). Only *APOE* ε4 status in the full cohort significantly impacted the Aβ ratio in the full cohort (median_APOE4+_  = 0.094, median_APOE4-_ = 0.11, *P* < 0.001) (Additional file 1: Table 4) but not the Aβ-positive group (*P* = 0.47) (Additional file 1: Table 2).

Finally, in the Aβ-positive group, both sAPPα (median_expansion+_ = 83 ng/mL, *P* = 0.047) and sAPPβ (median_expansion+_ = 82 ng/mL, *P* = 0.016) were significantly decreased in expansion carriers compared with non-carriers (median_ABCA7-_ = 113.5 ng/mL for sAPPα, median_ABCA7-_ = 117 ng/mL for sAPPβ) (Table 2, Fig. 1). In the clinical AD group, VNTR expansion carriers had significantly decreased sAPPβ levels compared to non-carriers (median_expansion+_  = 84.5 ng/mL, median_ABCA7-_ = 106.5 ng/mL, *P* = 0.041) (Additional file 1: Table 3, Additional file 2: Fig. 2); while, although tightly correlated with sAPPβ (*P* < 0.001, *r* = 0.96), sAPPα was not significantly changed in any of the groups (Additional file 1: Table 2). VNTR length sum as a continuous variable did not have a significant effect on either Aβ_1–42_, Aβ_1–40_, the Aβ ratio, sAPPα, or sAPPβ (Additional file 1: Tables 2 and 4 and Additional file 2: Figs. 1 and 3).

### CSF biomarkers indicative of tangle formation and neurodegeneration

In the cohort of carriers with in vivo evidence of amyloid burden, no changes were detected in either T-tau or P-tau_181_ levels (Table 2 and Fig. 1). In the clinically diagnosed AD cohort, an elevated P-tau_181_ concentration was observed in *APOE* ε4-positive carriers (median_APOE4+_  = 75 pg/mL) compared with *APOE* ε4-negative carriers (median_APOE4-_ = 62, *P* = 0.028) (Additional file 1: Table 4). Moreover, P-tau_181_ showed a weak significant increase in concentration with longer VNTR length (Additional file 1: Table 4, Additional file 2: Fig. 3), though there was no significant difference between expansion carriers and non-carriers. PTC carriers showed a significant increased concentration of T-tau (median_PTC+_  = 651.5 pg/mL) as opposed to non-carriers (median_ABCA7-_ = 442 pg/mL, *P* = 0.028) (Additional file 1: Table 3, Additional file 2: Fig. 2). For hFABP, having an *ABCA7* mutation or longer VNTR length was not associated with its concentration (Table 2 and Additional file 1: Table 3), while an increase in concentration was associated with an increase in age (*P* = 0.0058) and with males (*P* = 0.01) (Table 2, Additional file 1: Table 2) in both cohorts.

### CSF biomarkers of glial activation

AD patients showed a higher concentration of YKL-40 compared with control subjects (Table 1) and the concentration increased also with older age (*P* = 0.01) (Additional file 1: Table 2), while the concentration of YKL-40 was found to be decreased in VNTR expansion carriers as opposed to non-carriers (median_expansion+_  = 150 ng/mL, median_ABCA7-_ = 201 ng/mL, *P* = 0.0036, Table 2, Fig. 1). In comparison, median YKL-40 levels for controls were 153 ng/mL and thus similar to that of expansion carriers (Additional file 1: Table 1). VNTR length in the full and Aβ-positive cohort of non-PTC carriers was not associated with YKL-40 concentration (Additional file 1: Tables 2 and 4 and Additional file 2: Figs. 1 and 3).

## Discussion

To get a better understanding of the pathophysiology of dosage-reducing mutations in Alzheimer’s risk gene *ABCA7*, we studied nine different CSF biomarkers, representing several AD-associated pathways in our study cohort. We found that *ABCA7* VNTR expansion carriers have decreased levels of Aβ_1–42_, reduced Aβ_1–40_, sAPPα, sAPPβ, and YKL-40 levels compared with non-carrier AD patients. PTC carriers alone had increased T-tau levels compared with non-carriers, while P-tau_181_ levels rise with longer VNTR length, but this was only significant in the clinically diagnosed cohort. Overall, our results suggest that reduced *ABCA7* expression, or other mechanisms affected by these mutations, might influence APP processing and neurodegeneration and lead to a reduced inflammatory response to damage.

The biomarkers included in this study were selected based on in vitro evidence of possible functions of ABCA7. Biomarkers that were included were Aβ_1–42_ and Aβ_1–42_/Aβ_1–40_ ratio, which are typically lower in AD patients [13, 16]; Aβ_1–40_, which is slightly decreased in AD patients in a meta-analysis [13]; sAPPα and sAPPβ, which do not have a clear correlation with AD [13], and T-tau, P-tau_181_, YKL-40, and hFABP that are all moderately increased in patients [13, 16].

### Mutation carriers might be at increased risk of dysregulated APP processing

Amyloid pathology has often been implied in relation to ABCA7 function in both in vitro and in vivo studies [20–24]. A decrease or knock-out (KO) of *ABCA7* in mice, for example, led to increased Aβ-load or plaque burden in several studies [20, 21, 23], whilst the knock-out in macrophages and microglia led to reduced Aβ_1–42_ and Aβ_1–40_ uptake [22, 24]. Both impaired phagocytosis and elevated APP metabolism, whether or not influenced by changes in lipid metabolism, and caused by changes in ABCA7 dosage, have been suggested as possible causal mechanisms in AD [10, 32]. Moreover, several studies have found associations between common *ABCA7* GWAS SNPs and decreased Aβ_1–42_ CSF levels or increased amyloidosis using imaging biomarkers [33–35]. Additionally, analysis performed by our lab found *ABCA7* pLOF mutation carriers to be at increased odds of having abnormal Aβ_1–42_ CSF levels and found its levels also decreasing with increasing VNTR length [9, 36]. Our current findings in *ABCA7* repeat expansion carriers are in line with these observations. Aβ-ratio has been suggested to be a better measure of amyloid burden and more accurate to identify AD patients compared to Aβ_1–42_ alone, as it corrects for inter-individual variation in amyloid metabolism [15, 37]. In our data, only Aβ_1–42_ was significantly altered in mutation and expansion carriers, but not the ratio. One possible explanation might be that our cohort is too small to detect statistical differences, as the effect size of the Aβ_-_ratio is smaller than for Aβ_1–42_. However, another reason could be that, in contrast to in vitro and in vivo findings of increased plaque load with reduced ABCA7 [20, 21, 23], these expression-reducing mutations do not impact amount of plaques. In the group of expansion carriers, not only Aβ_1–42_ levels were reduced but there also was a reduction of other amyloid markers: Aβ_1–40_, sAPPα, and sAPPβ. This might be indicative for a downregulation of these peptides or dysregulation of overall APP processing in expansion carriers.

Similar to ABCA7, ApoE also has functions in lipid homeostasis and influences amyloid burden [38, 39]. Lipidated ApoE stimulates Aβ clearance, in which the ApoE4 variant is less effective than ApoE3 or ApoE2, and thus results in higher AD risk [40]. CSF Aβ_1–42_ levels are lower in *APOE* ε4 carriers compared with ε3 carriers, both preclinically and after onset of symptoms [41, 42]. Protein–protein interactions between ApoE and ABCA7 have been proposed before, as previous research suggests that *ABCA7* expression impacts lipidation of ApoE, and in this way might influence its effect on amyloid clearance [43]. We saw a decrease of Aβ_1–42_ levels in both *APOE ε4-*positive and *APOE ε4*-negative carriers when carrying an *ABCA7* mutation, but this decrease was especially pronounced in ε4-negative carriers. Possibly having an *ABCA7* mutation, and thus less lipidation, has less impact on amyloid clearance, as the ApoE4 protein is already severely impaired. However, there were no significant changes in Aβ ratio levels, implying no differences in amyloid deposition in both *APOE* and *ABCA7* mutation carrier. Alternatively, as both APOE and ABCA7 have been indicated to impact APP processing, these possible interactions could also impact APP metabolism, where again effect of ABCA7 dysregulation is clearer when APOE has not been impacted [32, 44].

Although in a meta-analysis no difference between AD patients and controls was found for APP products sAPPα and sAPPβ, we did see decreases for both sAPP fragments in AD patients in our cohort [13]. The function and involvement of the sAPP fragments in the brain is not yet known. Fragment sAPPα has been suggested to have a neuroprotective effect, whilst this is not true for sAPPβ [45, 46]. A decrease of sAPPα could therefore have detrimental effects. We also saw a decrease of sAPPα and sAPPβ in *ABCA7* VNTR expansion carriers in the Aβ-positive cohort. This in in contrast with earlier in vitro studies that reported that an increase of ABCA7 led to reduced levels of secreted sAPPα and sAPPβ, while suppressing ABCA7 with siRNA led to increased secretions of sAPPβ [23, 47]. As both are peptides formed during APP metabolism, sAPPα during the non-amyloidogenic pathway, and sAPPβ during the amyloidogenic pathway, a reduction also supports the hypothesis that APP processing is disrupted in expansion carriers. It further indicates that this reduced APP processing might be caused by a different mechanism than lower *ABCA7* expression, as these findings were not found in PTC carriers.

### T-tau is changed in PTC carriers and P-tau_181_ increases with VNTR length

Although a possible relationship between *ABCA7* and amyloid burden has been widely studied and established, this is not the case for NFT formation. Neurodegeneration, and thus T-tau levels, could be impacted through ABCA7’s possible role in inflammation or plaque burden. Previous research has also shown contrasting results when it came to CSF P-tau_181_ or T-tau and *ABCA7* mutations. Studies that investigated common (GWAS) *ABCA7* SNPs and its relation to either of these biomarkers have found both no association [35, 48] or an association [34, 49] in different studies. A genome-wide meta-analysis of CSF biomarker levels and a whole-exome rare-variant analysis both pointed to a connection between *ABCA7* and CSF P-tau_181_ levels, with the latter also demonstrating increased T-tau levels in *ABCA7* pLOF mutation carriers [36, 50]. This is in line with our finding of increased T-tau levels in PTC mutation carriers in the clinically diagnosed cohort. We also identified significantly increased P-tau_181_ levels along increasing VNTR length.

hFABP is a biomarker for neuronal degeneration and has been implicated in astrogliosis and vascular dysregulation too. It did not significantly alter in mutation carriers or along VNTR length, in contrast to T-tau. We did observe elevated hFABP with increasing age and in males, something which has been noted before [51, 52].

### Expansion carriers have lower YKL-40 levels indicative of gliosis

The role of the immune system in AD is still a topic of research and discussion. It seems that both an overstimulation, leading to excessive neuroinflammation, and an oppression, resulting in for example decreased phagocytosis of apoptotic cells and Aβ, are detrimental in the disease [53]. Moreover, the role of the immune system could also change throughout the disease [53]. ABCA7 has been implicated to play a role in inflammation and is expressed by both microglia and astrocytes [Accessed via celltypes.org/brain, Human Protein Atlas and Allen Brain Atlas in September 2023] [4, 24, 54–56]. YKL-40 is a marker of activated astrocytes, and in lesser extent, microglia was found to be moderately increased in AD patients and is widely reported as a marker of inflammation [57]. The biological role of YKL-40 is still unresolved but it is released by glial cells and has been suggested to be a pro-inflammatory molecule and have neuroprotective functions [57, 58]. In our cohort, we saw a significant reduction of YKL-40 levels in carriers of an expanded VNTR when compared to non-carrier AD patients. The levels of YKL-40 were even similar to that of healthy controls. An observation which was not mirrored in the PTC carriers, where we saw an, albeit not significant, increase in YKL-40 compared with non-carrier AD patients. Our results suggest that glial activation, typically seen in AD patients as response to AD neuropathology, is decreased in expansion carriers, which possibly reflects a reduced inflammatory response. Arguably the expansion has an impact on glial function, independent of the effect of ABCA7 dosage, resulting in this reduction. Previously, we identified an increase of *ABCA7* exon 19 skipping in individuals with longer VNTR length, which would result in a deletion of part of the first nucleotide-binding domain of the ABCA7 protein [9]. Possible formation of an ABCA7 protein without this domain impairs glial function and thus inflammation. Follow-up studies using additional inflammation markers would be useful to fully determine whether inflammatory response is impaired in expansion carriers. Finally, increasing age had a significant effect on (increasing) YKL-40 levels. This is also in line with earlier research and could reflect that either glial activation increases with age and/or that YKL-40 is a marker of a process that occurs in normal aging, but is exacerbated in AD [59, 60].

### Biomarker discrepancies between different mutation groups

Although both PTC mutations and the VNTR expansion have been associated with reduced *ABCA7* expression, we see a much more pronounced effect in the CSF of expansion carriers for some biomarkers, like Aβ_1–42_ [5, 9]. One possible explanation could be the presence of nonsense-mediated decay (NMD) escape and rescue splicing, splicing that could rescue the effect of the mutation in PTC carriers [7]. These two phenomena were observed in varying degrees, and could increase *ABCA7* expression in PTC mutation carriers, despite carrying a mutation, and thus attenuate the phenotype [7]. Only significant findings for expansion carriers remained in the Aβ-positive cohort, further suggesting that having a VNTR expansion has a bigger impact on pathophysiology. As mentioned earlier, it could be that for some biomarkers, a process other than *ABCA7* reduction impacts the pathophysiology reflected by the biomarkers. One possible pathophysiological mechanism in expansion carriers is exon 19 skipping and thus loss of a part of a crucial domain of the protein. Sample size could also influence our findings. Despite having access to a unique cohort of *ABCA7* mutation carriers, we still have a relatively small cohort of PTC mutation (*n* = 28) or expansion carriers (*n* = 16) of whom CSF is available, which limits statistical power, especially for those biomarkers with only moderate effect sizes.

Of the mutation carriers, seven samples were Aβ-negative, among these four PTC carriers, two expansion carriers and one sample with both an expansion and PTC mutation (Additional file 1: Table 5). PTC carriers with a negative amyloid classification had the p.E709fs or p.W1336* mutation, two mutations that we previously found to have potential transcript rescue mechanisms [7]. These are splicing events that could rescue the effect of the PTC mutation by for example in-frame exon skipping (Additional file 1: Table 5). The individual with both a PTC and expansion mutation had the c.5570 + 5G > C variant, which was not significantly associated with AD in our Belgian cohort [5], but was found to be significant in other studies [8]. Moreover, all of these samples were *APOE* ε4-negative. In the group of expansion carriers with Aβ-negative status, the average length of the VNTR sum was lower as compared with those that were Aβ-positive (mean_Aβ-_ = 9540 bp, mean_Aβ+_  = 10,194.7 bp). Only one out of three amyloid-negative samples had an *APOE* ε4 allele. Perhaps these differences can partially explain a possible milder phenotype, later AAO or higher Aβ_-_ratio levels in the Aβ-negative samples.

### Limitations of the study

The studied *ABCA7* mutations are relatively rare, limiting our cohort size and power to find differences between carriers and non-carriers. We did our analysis both in the full cohort of clinically diagnosed AD patients and in only those individuals with an Aβ-positive status based on the CSF Aβ ratio. The latter removed possible bias if subjects (also) had other syndromes, but also created a smaller cohort with less power to detect differences. In the Aβ-positive cohort, significant findings were only found for VNTR expansion carriers in YKL-40, Aβ_1–42_, Aβ_1–40_, sAPPα, and sAPPβ values. In the full cohort, all but the sAPPα finding remained, and additional changes in Aβ_1–42_, T-tau, and P-tau_181_ levels were detected in mutation carriers, PTC carriers, and along VNTR length, respectively. Another limitation of the study is not having access to longitudinal data, allowing us to follow up biomarker changes. In the future, it could be interesting to see how biomarkers progress between preclinical and symptomatic stages of mutation carriers. Replication of these results is warranted; however, due to the rare occurrence of these mutations, and absence of VNTR length data in public databases, other datasets with similar data lack power. But based on biological evidence, and biomarker evidence with common *ABCA7* SNPs, as discussed before, our results are in line with earlier findings.

## Conclusion

In conclusion, this study was the first CSF biomarker analysis of rare *ABCA7* mutation carriers and provides insight into the pathophysiology occurring in AD patients with those mutations. For the VNTR expansion carriers in the Aβ-positive, we found decreased levels of CSF Aβ_1–42_, Aβ_1–40_, sAPPα, sAPPβ, and YKL-40 levels compared to non-carriers, which are suggestive of reduced APP processing and inflammation, respectively. In the full cohort, CSF Aβ_1–42_ levels were decreased in mutation carriers too. Finally, in this cohort, T-tau levels were elevated in PTC mutation carriers whilst P-tau_181_ went up with longer VNTR length. These findings may inform both therapeutic strategy and clinical trial design and suggest that other mechanisms, beside lower *ABCA7* expression, might be causing some of the neuropathological changes in carriers.

### Supplementary Information

**Additional file 1: Table 1.** Full study cohort’s demographics and biomarker levels. **Table 2.** CSF biomarker associations for age, sex, VNTR length and APOE status in the Aβ-positive AD cohort. **Table 3.** Associations between CSF biomarkers and ABCA7 mutation status in the full cohort. **Table 4.** CSF biomarker associations for age, sex, VNTR length and APOE status in the full cohort. **Table 5.** Clinical data on ABCA7 mutation carriers included in the study.

**Additional file 2: Figure 1.** CSF biomarker level along VNTR length sum in the Aβ-positive AD cohort. Scatterplots showing biomarker concentrations (y-axis) of Aβ_1–42_ (A), Aβ_1–40_ (B), Aβ ratio (C), YKL-40 (D), sAPPα (E), sAPPβ (F), T-tau (G), P-tau_181_ (H) and hFABP (I), along the length of the sum of the VNTR alleles (x-axis, bp) in the Aβ-positive cohort, without PTC carriers. A trendline is shown (green when not significant, orange when significant) with standard error (shaded area). Significance was assessed with a linear regression with age, sex and *APOE* ε4 data as covariates on INT transformed data. **Figure 2.** CSF biomarker levels in different study groups in the full cohort. Comparison of different groups of ABCA7 mutation carriers or non-carriers, in the clinical AD cohort, using boxplots, depicting median and IQR, for the untransformed biomarkers: Aβ1–42 (A), Aβ1–40 (B), Aβ ratio (C), YKL-40 (D), sAPPα (E), sAPPβ (F), T-tau (G), P-tau181 (H) and hFABP (I). Controls are cognitively healthy subjects, shown here only for reference of normal physiological levels, as they were not included in the linear regression. T-tau and P-tau181 not depicted for controls, as these analyses were performed in a different lab. A sample with both a PTC and VNTR expansion mutation is shown for both groups in orange. For this sample no T-tau or P-tau181 measurements were available. Linear regression on INT transformed data was performed between different groups with age, sex and APOE ε4 status as covariates. *P < 0.05, **P < 0.01. **Figure 3.** CSF biomarker level along VNTR length sum in full AD cohort. Scatterplots showing biomarker concentrations (y-axis) of Aβ_1–42_ (A), Aβ_1–40_ (B), Aβ ratio (C), YKL-40 (D), sAPPα (E), sAPPβ (F), T-tau (G), P-tau_181_ (H) and hFABP (I), along the length of the sum of the VNTR alleles (x-axis, bp) in the total AD cohort, without PTC carriers. A trendline is shown (green when not significant, orange when significant) with standard error (shaded area). Significance was assessed with a linear regression with age, sex and *APOE* ε4 data as covariates on INT transformed data. **Figure 4.** Aβ1–42 and Aβ ratio levels along APOE ε4 and ABCA7 carrier status in the full cohort. Median Aβ_1–42_ (a, b) and Aβ ratio (c, d) levels according to *APOE *ε4 status and further stratified for *ABCA7* mutation carrier status (a, c) or VNTR expansion carrier status (b, d) in the full cohort. Significance was assessed with a Mann-Whitney U test on untransformed data. **P < 0.01.

## Data Availability

The datasets used and/or analysed during the current study are available from the corresponding author on reasonable request.

## Abbreviations

GWAS: Genome-wide association study
AD: Alzheimer’s disease
PTC: Premature termination codon
ABCA7: ATP-binding cassette transporter family A member 7
VNTR: Variable number of tandem repeats
CAA: Cerebral amyloid angiopathy
CSF: Cerebrospinal fluid
Aβ: Amyloid-β total tau
T-tau: Total tau
P-tau_181_: Phosphorylated tau at threonine 181
CHI3L1/YKL-40: Chitinase-3-like protein 1
hFABP: Heart fatty acid binding protein
NFT: Neurofibrillary tangles
LP: Lumbar puncture
ELISA: Enzyme-linked immunosorbent assays
ECL: Electro chemiluminescent
MSD: Meso Scale Discovery
CV: Coefficient of variation
INT: Inverse normal transformation
SE: Standard errors
AAO: Age at onset
DD: Disease duration
KO: Knock-out
IQR: Interquartile range
