# Supplementary figures and images for: CSF biomarker analysis of ABCA7 mutation carriers suggests altered APP processing and reduced inflammatory response
Source: Alzheimers Res Ther. 2023 Nov 9;15:195. doi: 10.1186/s13195-023-01338-y (PMC10634183; doi:10.1186/s13195-023-01338-y)

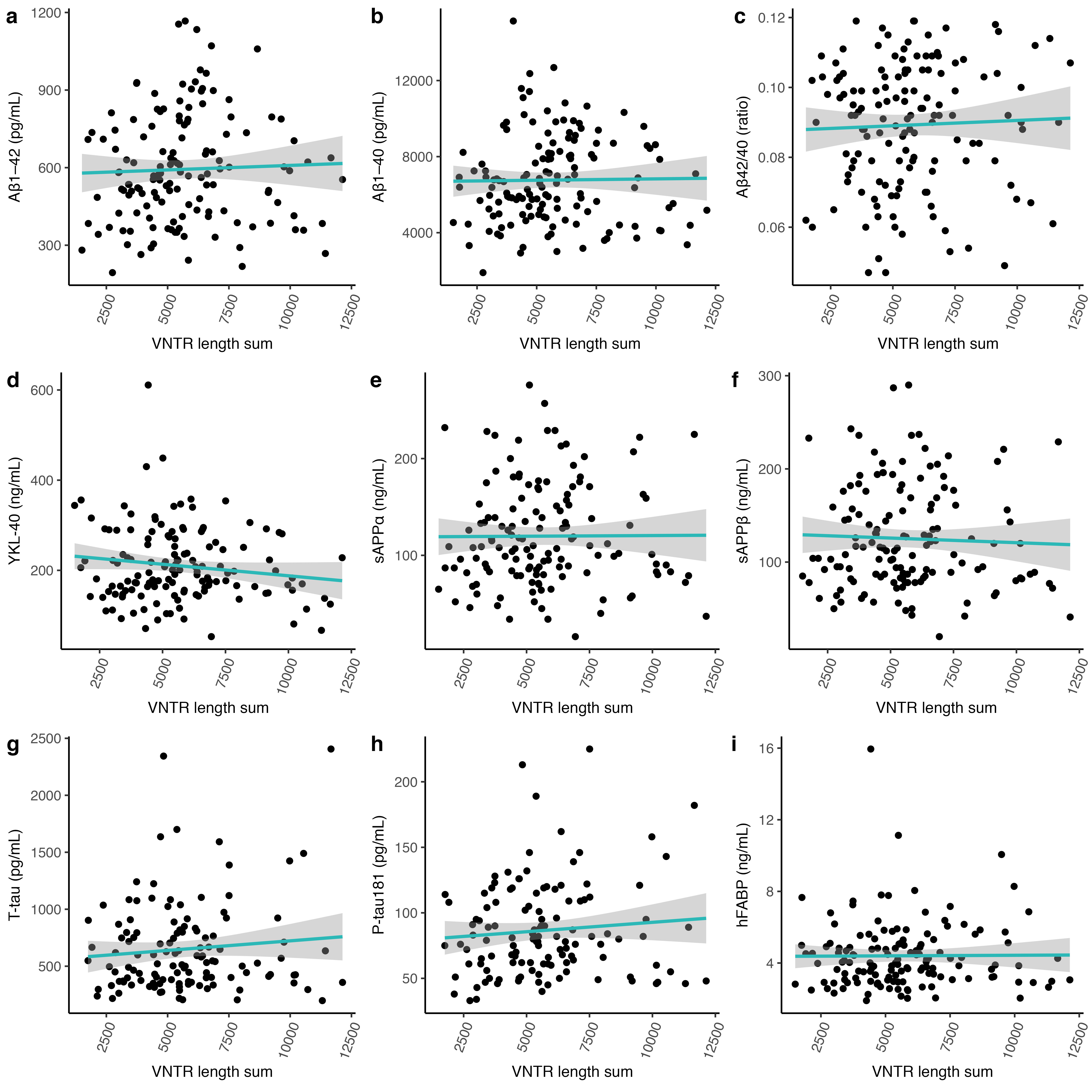

Supplement: Supplementary file 2 — Additional file 2: Figure 1. CSF biomarker level along VNTR length sum in the Aβ-positive AD cohort. Scatterplots showing biomarker concentrations (y-axis) of Aβ1–42 (A), Aβ1–40 (B), Aβ ratio (C), YKL-40 (D), sAPPα (E), sAPPβ (F), T-tau (G), P-tau181 (H) and hFABP (I), along the length of the sum of the VNTR alleles (x-axis, bp) in the Aβ-positive cohort, without PTC carriers. A trendline is shown (green when not significant, orange when significant) with standard error (shaded area). Significance was assessed with a linear regression with age, sex and APOE ε4 data as covariates on INT transformed data. Figure 2. CSF biomarker levels in different study groups in the full cohort. Comparison of different groups of ABCA7 mutation carriers or non-carriers, in the clinical AD cohort, using boxplots, depicting median and IQR, for the untransformed biomarkers: Aβ1–42 (A), Aβ1–40 (B), Aβ ratio (C), YKL-40 (D), sAPPα (E), sAPPβ (F), T-tau (G), P-tau181 (H) and hFABP (I). Controls are cognitively healthy subjects, shown here only for reference of normal physiological levels, as they were not included in the linear regression. T-tau and P-tau181 not depicted for controls, as these analyses were performed in a different lab. A sample with both a PTC and VNTR expansion mutation is shown for both groups in orange. For this sample no T-tau or P-tau181 measurements were available. Linear regression on INT transformed data was performed between different groups with age, sex and APOE ε4 status as covariates. *P < 0.05, **P < 0.01. Figure 3. CSF biomarker level along VNTR length sum in full AD cohort. Scatterplots showing biomarker concentrations (y-axis) of Aβ1–42 (A), Aβ1–40 (B), Aβ ratio (C), YKL-40 (D), sAPPα (E), sAPPβ (F), T-tau (G), P-tau181 (H) and hFABP (I), along the length of the sum of the VNTR alleles (x-axis, bp) in the total AD cohort, without PTC carriers. A trendline is shown (green when not significant, orange when significant) with standard error (shaded area). Sig [file 13195_2023_1338_MOESM2_ESM.zip › AdditionalFigure1_ART_LenaDuchateau.png]

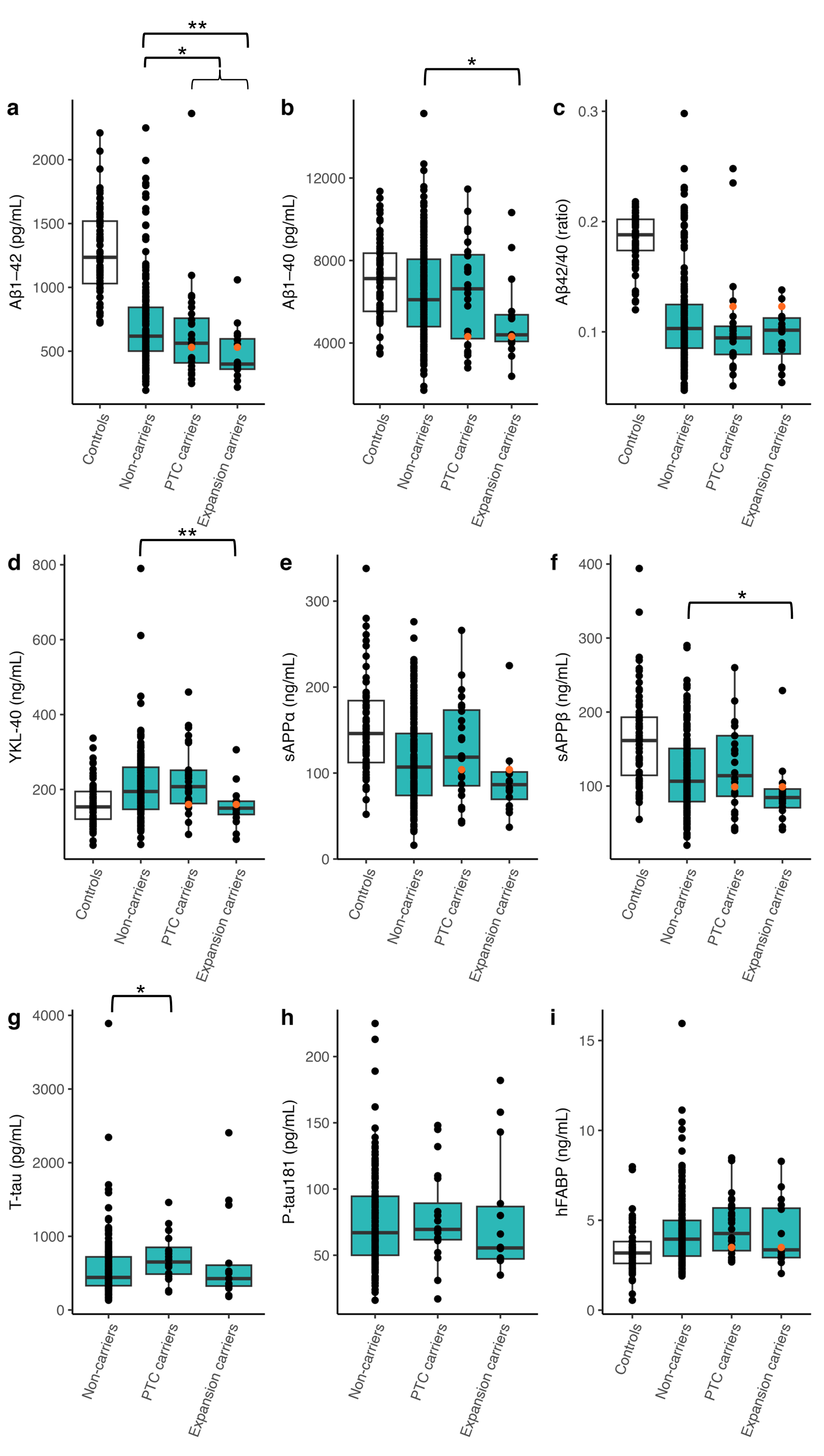

Supplement: Supplementary file 2 — Additional file 2: Figure 1. CSF biomarker level along VNTR length sum in the Aβ-positive AD cohort. Scatterplots showing biomarker concentrations (y-axis) of Aβ1–42 (A), Aβ1–40 (B), Aβ ratio (C), YKL-40 (D), sAPPα (E), sAPPβ (F), T-tau (G), P-tau181 (H) and hFABP (I), along the length of the sum of the VNTR alleles (x-axis, bp) in the Aβ-positive cohort, without PTC carriers. A trendline is shown (green when not significant, orange when significant) with standard error (shaded area). Significance was assessed with a linear regression with age, sex and APOE ε4 data as covariates on INT transformed data. Figure 2. CSF biomarker levels in different study groups in the full cohort. Comparison of different groups of ABCA7 mutation carriers or non-carriers, in the clinical AD cohort, using boxplots, depicting median and IQR, for the untransformed biomarkers: Aβ1–42 (A), Aβ1–40 (B), Aβ ratio (C), YKL-40 (D), sAPPα (E), sAPPβ (F), T-tau (G), P-tau181 (H) and hFABP (I). Controls are cognitively healthy subjects, shown here only for reference of normal physiological levels, as they were not included in the linear regression. T-tau and P-tau181 not depicted for controls, as these analyses were performed in a different lab. A sample with both a PTC and VNTR expansion mutation is shown for both groups in orange. For this sample no T-tau or P-tau181 measurements were available. Linear regression on INT transformed data was performed between different groups with age, sex and APOE ε4 status as covariates. *P < 0.05, **P < 0.01. Figure 3. CSF biomarker level along VNTR length sum in full AD cohort. Scatterplots showing biomarker concentrations (y-axis) of Aβ1–42 (A), Aβ1–40 (B), Aβ ratio (C), YKL-40 (D), sAPPα (E), sAPPβ (F), T-tau (G), P-tau181 (H) and hFABP (I), along the length of the sum of the VNTR alleles (x-axis, bp) in the total AD cohort, without PTC carriers. A trendline is shown (green when not significant, orange when significant) with standard error (shaded area). Sig [file 13195_2023_1338_MOESM2_ESM.zip › AdditionalFigure2_ART_LenaDuchateau.png]

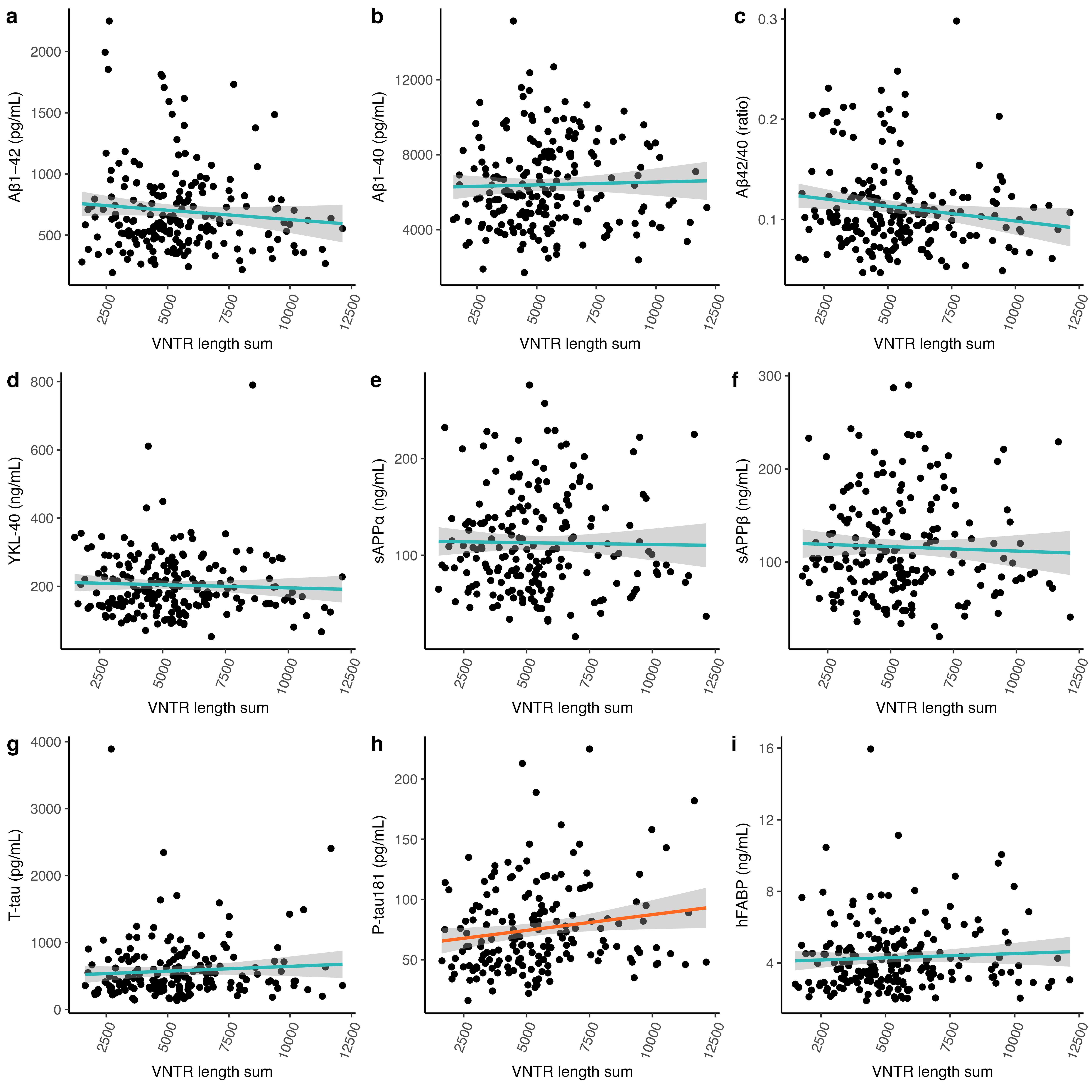

Supplement: Supplementary file 2 — Additional file 2: Figure 1. CSF biomarker level along VNTR length sum in the Aβ-positive AD cohort. Scatterplots showing biomarker concentrations (y-axis) of Aβ1–42 (A), Aβ1–40 (B), Aβ ratio (C), YKL-40 (D), sAPPα (E), sAPPβ (F), T-tau (G), P-tau181 (H) and hFABP (I), along the length of the sum of the VNTR alleles (x-axis, bp) in the Aβ-positive cohort, without PTC carriers. A trendline is shown (green when not significant, orange when significant) with standard error (shaded area). Significance was assessed with a linear regression with age, sex and APOE ε4 data as covariates on INT transformed data. Figure 2. CSF biomarker levels in different study groups in the full cohort. Comparison of different groups of ABCA7 mutation carriers or non-carriers, in the clinical AD cohort, using boxplots, depicting median and IQR, for the untransformed biomarkers: Aβ1–42 (A), Aβ1–40 (B), Aβ ratio (C), YKL-40 (D), sAPPα (E), sAPPβ (F), T-tau (G), P-tau181 (H) and hFABP (I). Controls are cognitively healthy subjects, shown here only for reference of normal physiological levels, as they were not included in the linear regression. T-tau and P-tau181 not depicted for controls, as these analyses were performed in a different lab. A sample with both a PTC and VNTR expansion mutation is shown for both groups in orange. For this sample no T-tau or P-tau181 measurements were available. Linear regression on INT transformed data was performed between different groups with age, sex and APOE ε4 status as covariates. *P < 0.05, **P < 0.01. Figure 3. CSF biomarker level along VNTR length sum in full AD cohort. Scatterplots showing biomarker concentrations (y-axis) of Aβ1–42 (A), Aβ1–40 (B), Aβ ratio (C), YKL-40 (D), sAPPα (E), sAPPβ (F), T-tau (G), P-tau181 (H) and hFABP (I), along the length of the sum of the VNTR alleles (x-axis, bp) in the total AD cohort, without PTC carriers. A trendline is shown (green when not significant, orange when significant) with standard error (shaded area). Sig [file 13195_2023_1338_MOESM2_ESM.zip › AdditionalFigure3_ART_LenaDuchateau.png]

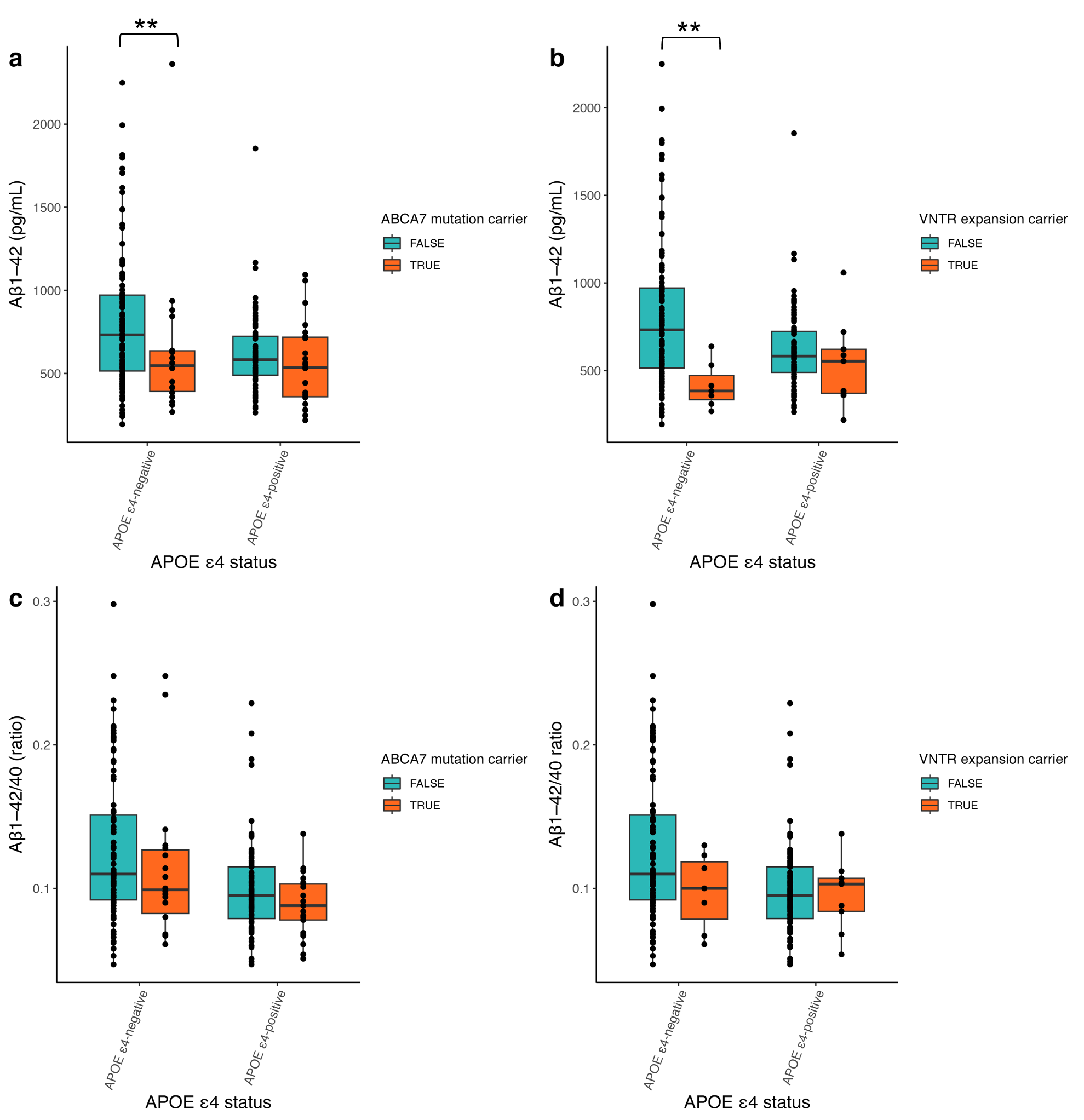

Supplement: Supplementary file 2 — Additional file 2: Figure 1. CSF biomarker level along VNTR length sum in the Aβ-positive AD cohort. Scatterplots showing biomarker concentrations (y-axis) of Aβ1–42 (A), Aβ1–40 (B), Aβ ratio (C), YKL-40 (D), sAPPα (E), sAPPβ (F), T-tau (G), P-tau181 (H) and hFABP (I), along the length of the sum of the VNTR alleles (x-axis, bp) in the Aβ-positive cohort, without PTC carriers. A trendline is shown (green when not significant, orange when significant) with standard error (shaded area). Significance was assessed with a linear regression with age, sex and APOE ε4 data as covariates on INT transformed data. Figure 2. CSF biomarker levels in different study groups in the full cohort. Comparison of different groups of ABCA7 mutation carriers or non-carriers, in the clinical AD cohort, using boxplots, depicting median and IQR, for the untransformed biomarkers: Aβ1–42 (A), Aβ1–40 (B), Aβ ratio (C), YKL-40 (D), sAPPα (E), sAPPβ (F), T-tau (G), P-tau181 (H) and hFABP (I). Controls are cognitively healthy subjects, shown here only for reference of normal physiological levels, as they were not included in the linear regression. T-tau and P-tau181 not depicted for controls, as these analyses were performed in a different lab. A sample with both a PTC and VNTR expansion mutation is shown for both groups in orange. For this sample no T-tau or P-tau181 measurements were available. Linear regression on INT transformed data was performed between different groups with age, sex and APOE ε4 status as covariates. *P < 0.05, **P < 0.01. Figure 3. CSF biomarker level along VNTR length sum in full AD cohort. Scatterplots showing biomarker concentrations (y-axis) of Aβ1–42 (A), Aβ1–40 (B), Aβ ratio (C), YKL-40 (D), sAPPα (E), sAPPβ (F), T-tau (G), P-tau181 (H) and hFABP (I), along the length of the sum of the VNTR alleles (x-axis, bp) in the total AD cohort, without PTC carriers. A trendline is shown (green when not significant, orange when significant) with standard error (shaded area). Sig [file 13195_2023_1338_MOESM2_ESM.zip › AdditionalFigure4_ART_LenaDuchateau.png]
